# *TP53* and *PIK3CA* gene mutations in adenocarcinoma, squamous cell carcinoma and high-grade intraepithelial neoplasia of the cervix

**DOI:** 10.1186/s12967-014-0255-5

**Published:** 2014-09-16

**Authors:** Maria Lina Tornesello, Clorinda Annunziata, Luigi Buonaguro, Simona Losito, Stefano Greggi, Franco M Buonaguro

**Affiliations:** Molecular Biology and Viral Oncology Unit, Istituto Nazionale Tumori “Fond Pascale” - IRCCS – Via Mariano Semmola, 80131 Napoli, Italy; Pathology Department, Istituto Nazionale Tumori “Fond Pascale” - IRCCS – Via Mariano Semmola, 80131 Napoli, Italy; Uro-gynecology Department, Istituto Nazionale Tumori “Fond Pascale” - IRCCS – Via Mariano Semmola, 80131 Napoli, Italy

**Keywords:** *TP53* gene, *PIK3CA* gene, Cervix, Squamous cell carcinoma, Adenocarcinoma, Cervical intraepithelial neoplasia

## Abstract

**Background:**

Mutations in the tumor suppressor gene *TP53* and proto-oncogene *PIK3CA* and alterations of p53 and *PIK3CA* AKT mTOR pathways are common events in several human cancers. We focused on the analysis of *TP53* and *PIK3CA* gene variations in adenocarcinoma, squamous cell carcinoma as well as in intraepithelial neoplasia grade 3 of the cervix.

**Methods:**

DNA samples from 28 cervical adenocarcinoma, 55 squamous cell carcinoma and 31 intraepithelial neoplasia grade 3 (CIN3), previously characterized in terms of human papillomavirus (HPV) prevalence and genotype distribution, were analyzed for *TP53* and *PIK3CA* mutations in the exons 4–9 and exon 9, respectively.

**Results:**

Single nucleotide substitutions in *TP53* and *PIK3CA* genes were detected in 36% and 11% of adenocarcinoma, in 16% and in 5% of squamous cell carcinoma, and in 13% and none of CIN 3, respectively. Nucleotide changes in *TP53* were significantly more frequent in adenocarcinoma cases than in squamous cell carcinoma and CIN3 (P = 0.035) and were independent from HPV infection status.

**Conclusions:**

Mutations in the *TP53* gene and to lesser extent in the *PIK3CA* gene seem more frequent in cervical adenocarcinoma than in squamous cell carcinoma and CIN3. Whether *TP53* and *PIK3CA* gene mutations have an impact on prognosis and response to molecularly targeted therapies as well as in cytotoxic drugs in different cervical cancer histotypes needs to be analyzed in investigative clinical trials.

## Introduction

Cervical cancer is the fourth most common cancer diagnosed in women worldwide with an estimated 527,624 new cases and 265,653 deaths in 2012 [1]. The major histopathologic types are squamous cell carcinoma and adenocarcinoma which constitute about 85% and 10-12% of all cases of cervical cancer, respectively. The squamous cell carcinoma arises in the squamocolumnar junction between the ectocervical squamous epithelium and the endocervical columnar epithelium and is preceded by a long phase of cervical intraepithelial neoplasia (CIN1, CIN2 and CIN3) [2]. The adenocarcinoma originates from glandular precursor lesions of the endocervical mucosa and comprises several histological subtypes such as the mucinous adenocarcinoma (intestinal, endocervical or signet-ring), the endometrioid and non-mucinous adenocarcinoma (clear cell, serous) [3].

Oncogenic HPVs, mainly HPV 16 and 18 genotypes, have been strongly associated with the risk to develop intraepithelial lesions, squamous cell carcinoma and adenocarcinoma of the cervix [4]. However, the majority of HPV infections induce low grade squamous epithelial lesions that in more than 90% of cases spontaneously regress and in about 10% become transforming infections, characterized by several molecular changes [5]. The early genes E6 and E7 of high risk HPVs are consistently expressed in HPV-related cancers and derived tumor cell lines and contribute to the transformation of infected epithelial cells mainly through the inactivation of p53 and pRb oncosuppressors and related pathways [6]. However, the constitutive expression of early viral genes is not in itself sufficient to induce and maintain the transformation status and accumulation of genetic and/or epigenetic alterations over time may be crucial for the ultimate progression to cancer [5,7,8].

A number of studies have demonstrated that adenocarcinoma has worse prognosis with higher rates of metastases and decreased survival compared with squamous cell carcinoma [9]. However, few studies have examined whether distinct molecular profiles underlie the pathogenesis of the two types of cervical cancer.

Several cellular genes such as *TP53* [10,11], *PIK3CA* [12], c-Myc (Myc) and ErbB2 [13], cIAP1 [14], Ras [15], PTEN [16] and LKB1 [17] have been found mutated or functional inactivated in variable proportions of cervical cancers. Comprehensive analysis of genomic aberrations in cervical tumors allowed to identify, besides the previously characterized mutations in *TP53* and *PIK3CA* genes, unknown mutations in MAPK1, HLA-B, EP300, FBXW7, NFE2L2, and ERBB2 genes in squamous cell carcinoma and somatic mutations of ELF3 (13%) and CBFB (8%) genes in adenocarcinomas [18].

Mutations in *TP*53 gene are among the most common genetic alterations in many human malignancies [19-23]. Up to 90% of *TP*53 mutations are non-synonymous and determine single amino-acid changes primarily within the DNA binding domain region (exon 5–8) located between codons 125 and 300 [24]. In a recent meta-analysis, including 1353 cervical tumors the frequency of non-synonymous mutations in the DNA-binding domain of the *TP53* gene was found significantly higher in adenocarcinoma (32 of 241; 13.3%) compared to squamous cell carcinoma (39 of 657; 5.9%; P = 0.0003, χ^2^ test), [10]. The proportion of adenocarcinoma with mutated *TP53* varied from 4% in North America to 19% in Asia. Among the six hot-spot codons of *TP53* gene only three codons (175, 248 and 273) were found commonly mutated in both types of cervical cancer. No study, however, has systematically analyzed the frequency of *TP53* mutations in different histological types of adenocarcinoma, in squamous cell carcinoma and in pre-invasive neoplastic lesions of the cervix [10].

A number of studies reported that the phosphoinositide-3-kinase-catalytic-alpha (*PIK3CA*) gene is frequently mutated in the helical domain within exon 9 (codons 542 and 545) and in the kinase domain within exon 20 (codon 1047) of several types of human cancer [25]. In cervical cancer activating mutations in *PIK3CA* gene have been found almost exclusively in exon 9 [18,26-28]. Knowledge of mutational status of *PIK3CA* gene is particularly relevant considering that several anticancer drugs, targeting PI3K/Akt pathway, have given promising preliminary results in human malignancies [29]. McIntyre *et al.* (2013) have recently reported that in cervical cancer patients treated with radical chemoradiotherapy the *PIK3CA* mutation status was strongly associated with overall survival in FIGO stage IB/II but not stage III/IVA [26]. In addition, *PIK3CA* mutations in patients with advanced breast, ovarian, endometrial, and cervical cancers have been found associated with a higher response rate to treatments that include PI3K/AKT/mTOR inhibitors [30]. These observations suggest that *PIK3CA* could represent a potential drug targetable molecule for the treatment of cervical cancer.

We performed a retrospective study investigating the frequency of *TP53* and *PIK3CA* mutations in pre-treatment biopsies from a cohort of patients with mucinous and non-mucinous adenocarcinoma, squamous cell carcinoma and CIN 3 and we correlated the *TP53* and *PIK3CA* mutational status with histological subtypes and HPV status.

## Materials and methods

### Patient and tissue samples

One hundred and fourteen formalin-fixed and paraffin-embedded cervical neoplasia biopsies from patients referred to the Gynecology Unit at the National Cancer Institute Fond Pascale, from 2002 to 2008, were included in the study. All cases have been previously characterized in terms of histology, DNA quality and HPV genotypes [31,32]. In particular, from each paraffin block, an initial 10-μm thick section was obtained for hematoxylin–eosin staining, followed by four 10 μm sections that were collected in separate sterile Eppendorf tubes for PCR analysis. Slides immediately adjacent to the tissue section used for gene mutation analysis were reviewed by the pathologist to verify the presence of neoplastic tissue. This morphological check showed that the mean percentage of tumour cells staining was 64% (range 10–100%). Histological subtypes were determined in accordance to Young et al. [33] and to The Bethesda 2001 system. Tissues were graded as mucinous adenocarcinoma (endocervical and intestinal types, n = 12), non-mucinous adenocarcinoma (endometrioid, clear cell and serous types, n = 14), mixed adenocarcinoma (n = 2), invasive squamous cell carcinoma (n = 55) and CIN 3, according to the highest grade present within a lesion.

Genomic DNA was extracted according to published procedures [34]. In particular tissue samples were deparaffinized in xylenes and digested with Proteinase K (150 μg per ml at 60°C for 30 min) in 100 μl of lysis buffer (10 mM Tris–HCl pH 7.6, 5 mM EDTA, 150 mM NaCl, 1% SDS), followed by DNA purification by phenol and phenol-chloroform-isoamyl alcohol (25:24:1) extraction and ethanol precipitation in 0.3 M sodium acetate (pH 4.6). The study was approved by the institutional review board of the Istituto Nazionale Tumori Pascale.

### *TP53* codon 4–9 mutational analysis

The analysis of *TP53* gene in exons 4–9 was performed using specific oligonucleotides and amplification protocols according to the IARC guidelines (http://www-p53.iarc.fr/Download/TP53_DirectSequencing_IARC.pdf). All PCR reactions were undertaken using 10 to 100 ng genomic DNA in 50-μL reaction mixture following the IARC amplification procedures using in all reactions the Hot Master buffer and the Hot Master Taq DNA Polymerase (5 Prime GmbH, Hamburg, Germany). DNAs were amplified in a Perkin-Elmer GeneAmp PCR System 9700 thermal cycler. All samples with sufficient amount of DNA were subjected to bidirectional direct sequencing analysis by Primm Srl Laboratories (Milan, Italy).

### *PIK3CA* codon 9 mutational analysis

*PIK3CA* codon 9 was amplified by a seminested PCR using in the outer reaction the oligoprimer PIK3-9-F1 (5′- TGGTCTTGTT GTTGGCTAA) with PIK3-9-R1 (5′- CTTACCTGTGACTCCATAGAA), generating a 410 bp, and in the inner reaction the oligoprimer PIK3-9-F2 (5′- ACTATTCTGTGACTGGTGTAAT) with PIK3-9-R1, generating a 381 bp fragment encompassing the hot spot codons 542 and 545 and designed to avoid amplification of the *PIK3CA* pseudogene. PCR reactions were performed in 50 μL reaction mixture containing 50 to 100 ng of target DNA, 5 pmol of each primer, 2.5 mM MgCl2, 50 mM of each dNTP and 5 ul Hot Master buffer and 1 U of Hot Master Taq DNA Polymerase (5 Prime GmbH, Hamburg, Germany). DNA was amplified in a Perkin-Elmer GeneAmp PCR System 9700 thermal cycler with the following steps: an initial 1-min denaturation at 94°C, followed by 45 amplification cycles of 58°C for 30 sec, 72°C for 30 sec, 94°C for 30 sec and a 1-min final annealing at 58°C followed by 5-min elongation at 72°C. All samples were subjected to bidirectional direct sequencing analysis.

### Statistical analyses

The planned test for statistical evaluation were Fisher’s exact test, Yates corrected χ^2^ test or χ^2^ test for trend, as appropriate, to compare the proportions of cases mutated in *TP53* or *PIK3CA* genes among patients stratified by, age, tumour hystotype and HPV infection. All analyses were performed with Epi Info 6 Statistical Analysis System Software (6.04d, 2001, Centers for Disease Control and Prevention, USA). Differences were considered to be statistically significant when *P* values were less than 0.05.

## Results

A total of 114 patients with cervical adenocarcinoma, squamous cell carcinoma and CIN 3 were analyzed for the presence of mutations in exons 4–9 of *TP53* and exon 9 of *PIK3CA* genes (Table 1). Twenty-eight patients (25%) were diagnosed with adenocarcinoma carcinoma, 55 (48%) with squamous cell carcinoma and 31 (27%) with CIN 3. The prevalence of high risk HPV was 72% in adenocarcinoma, 85% in squamous cell carcinoma and 55% in CIN 3 cases. HPV16 was the most frequent viral genotype in all histological categories being present in 67%, 81% and 74% of HPV-related adenocarcinoma, squamous cell carcinoma and CIN 3, respectively [31,32].

**Table 1:** ***TP53*** **and** ***PIK3CA*** **mutations and tumor clinical characteristics (n = 114)**

	***TP53*** **mutant**		***TP53*** **wild type**		***P*** **value**	***PI3KCA*** **mutant**		***PIK3CA*** **wild type**		***P*** **value**
	**(n = 23)**		**(n = 91)**			**(n = 6)**		**(n = 108)**		
**Variable**	n	(%)	n	(%)						
**Mean age [SD]**	48.7	[±10.6]	51.4	[±14.7]	0.326	56.9	[±10.5]	50.9	[±14.5]	0.364
**Histology**					0.035*					0.400**
Adenocarcinoma (n = 28)	10	(36)	18	(64)		3	(11)	25	(89)	
Squamous carcinoma (n = 55)	9	(16)	46	(84)		3	(5)	52	(95)	
CIN 3 (n = 31)	4	(13)	27	(87)		0				
Grading***					0.603					0.664
G1 (n = 5)	0					0				
G2 (n = 23)	7	(30)	16	(70)		1	(4)	22	(96)	
G3 (n = 55)	12	(22)	43	(78)		5	(9)	50	(91)	
**HPV status**										
Negative (n = 37)	10	(27)	27	(73)		4	(11)	33	(89)	
Positive (n = 77)	13	(17)	64	(83)		2	(3)	75	(97)	

*χ^2^for trend = 4.44.

**Fisher exact test.

***Well differentiated (G1); Moderately differentiated (G2); Poorly differentiated carcinoma (G3).

**Figure 1:**
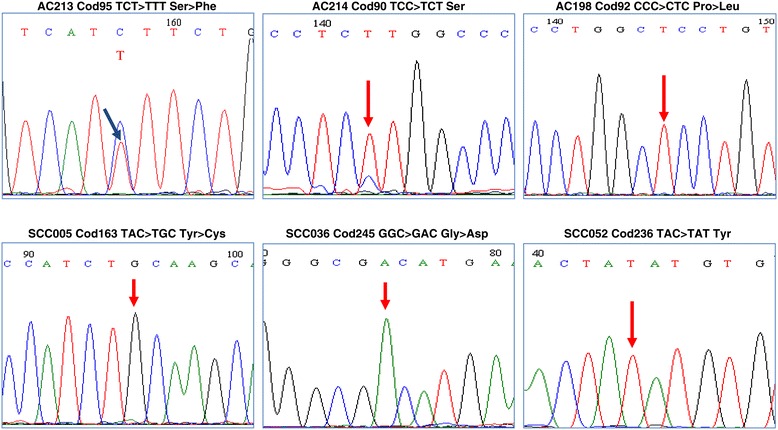
**DNA sequence electropherograms showing nucleotide mutations in exons 4–9 of** ***TP53*** **gene.** Representative examples of heterozygous mutations (sample AC213) and homozygous mutations (samples AC214, AC198, SCC005, SCC036, SCC052).

*TP53* gene was mutated in 10 out of 28 (36%) adenocarcinoma, in 9 out of 55 (16%) squamous cell carcinoma and in 4 out of 31 (13%) of CIN 3 cases. In particular 46%, 7.9% and 15.3% of HPV16-positive adenocarcinoma, squamous cell carcinoma and CIN3, respectively, harbored mutations in *TP53* gene. One out of nine (11.1%) squamous cell carcinoma positive for high risk HPVs other than type 16 was mutated in *TP53* gene. None of adenocarcinoma and CIN3 cases positive for high risk HPVs other than type 16 were mutated in the exons 4–9 of *TP53* gene. Among HPV-negative samples 50%, 75% and 7.1% of adenocarcinoma, squamous cell carcinoma and CIN3, respectively, were mutated in the *TP53*. The most common nucleotide changes were missense mutations (n = 17) affecting the DNA binding domain of p53 (Table 2). Interestingly, in 5 cases the single nucleotide substitution appeared as an homozygous mutation suggesting that major genetic alterations including loss of heterozygosity affected chromosome 17 (Figure 1). No insertion or deletions were detected. *TP53* mutations were significantly more frequent in adenocarcinoma (χ^2^ for trend =4.44, *P* = 0.035), mainly in the mucinous endocervical adenocarcinoma histotype (54%), compared to squamous cell carcinoma and CIN3 (Table 3). The combined mutational pattern of *TP53* and *PIK3CA* genes showed that a relevant proportion of nucleotide changes was represented by C:G > T:A transitions (39%) potentially arising from deamination of DNA bases.

**Table 2:** **Histological characteristics of cervical samples, HPV status,** *TP53* **(exons 4–9) and** ***PIK3CA*** **(exon 9) mutations**

**Patient no**	**Histology**	**HPV**	***TP53*** **Mut**		***PIK3CA*** **Mut**	
AC206	Mucinous Endocervical Adenocarcinoma	HPV16 E	T231N	C > A		
AC134	Mucinous Endocervical Adenocarcinoma	HPV16 E,18	M237K	T > A		
AC174	Mucinous Endocervical Adenocarcinoma	Neg	C258C	A > T		
			G295N	A > T		
AC213	Mucinous Endocervical Adenocarcinoma	HPV16 AA	S095F	C > T		
AC214	Mucinous Endocervical Adenocarcinoma	HPV16 E	S090S	C > T		
AC198	Mucinous Endocervical Adenocarcinoma	Neg	P092L	C > T	E547K	G > A
AC193	Mucinous Endocervical Adenocarcinoma	Neg	A307S	G > T	S541Y	C > A
AC201	Mucinous Endometrioid Adenocarcinoma	HPV16 E	E286E	A > G		
AC218	Mucinous Endometrioid Adenocarcinoma	HPV16 E			Q546Q	G > A
AC199	Non-mucinous Serous Adenocarcinoma	Neg	M237I	G > C		
AC210	Non-mucinous Serous Adenocarcinoma	HPV16	Y236C	A > G		
SCC005	Squamous Cell Carcinoma	Neg	Y163C	A > G		
SCC008	Squamous Cell Carcinoma	Neg	H296H	C > T		
SCC010	Squamous Cell Carcinoma	Neg	I162I	C > T		
SCC107	Squamous Cell Carcinoma	HPV16	int 14077	C > T		
SCC022	Squamous Cell Carcinoma	HPV16	P250T	C > A		
SCC032	Squamous Cell Carcinoma	HPV16	I255I	C > T		
SCC036	Squamous Cell Carcinoma	HPV45	G245D	G > A		
SCC045	Squamous Cell Carcinoma	Neg	S260T	T > A		
SCC052	Squamous Cell Carcinoma	Neg	Y236T	C > T		
SCC034	Squamous Cell Carcinoma	Neg			E545A	A > C
SCC006	Squamous Cell Carcinoma	HPV16			E545K	G > A
SCC019	Squamous Cell Carcinoma	Neg			D527N	G > A
SCC064	Squamous Cell Carcinoma	Neg	C242C	C > T		
CIN085	Cervical Intraepithelial Neoplasia 3	HPV16	V143A	T > C		
CIN090	Cervical Intraepithelial Neoplasia 3	Neg	Y95Y	C > T		
			R282W	C > T		
CIN099	Cervical Intraepithelial Neoplasia 3	HPV16	int 13305	C > T

**Table 3:** **Frequency of** ***TP53*** **and** ***PIK3CA*** **mutations in cervical adenocarcinoma according to histological subtypes**

	***TP53*** **Mut**		***PIK3CA*** **Mut**	
**Diagnosis**	**n**	**(%)**	**n**	**(%)**
*Mucinous adenocarcinoma*				
Endocervical (n = 11)	6	(54)	2	(18)
Intestinal (n = 1)	0		0	
*Endometrioid* (n = 8)	2	(25)	1	(12)
*Non-mucinous adenocarcinoma*				
Clear cell (n = 2)	0		0	
Serous (n = 4)	2	(40)	0	
*Mixed adenocarcinoma* (n = 2)*	0		0	
*All adenocarcinoma* (n = 28)	10	(36)	3	(11)

*Serous/Glassy cells Adenocarcinoma (n = 1); Intestinal/signet ring cell Adenocarcinoma (n = 1).

There was no statistically significant difference in *TP53* gene mutation frequency between HPV-positive and HPV-negative samples in all histological groups.

**Figure 2:**
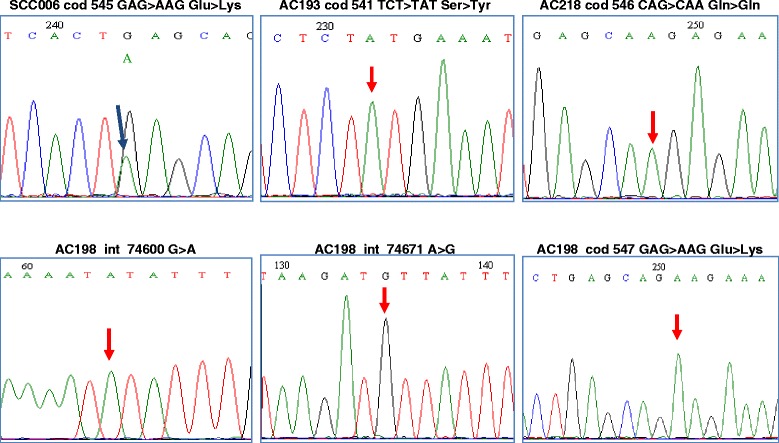
**DNA sequence electropherograms showing nucleotide mutations in exon 9 of** ***PIK3CA*** **gene.** Representative examples of heterozygous mutations (sample SCC006) and homozygous mutations (samples AC193, AC218, AC198).

The exon 9 of *PIK3CA* gene was found mutated in six (5%) out of 114 cases (Table 1). Non-synonymous mutations were identified in three (11%) of the adenocarcinomas at codons 541 (TCT > TAT, Ser > Tyr), 546 (CAG > CAA, Gln > Gln) and 547 (GAG > AAG, Glu > Lys), and in all three cases the sequence electropherograms showed homozygous mutations suggestive of major chromosomal aberrations affecting the 3q26.3 loci (Figure 2). Heterozygous mutations in *PIK3CA* gene were found in three cases (5%) of squamous cell carcinoma at codons 527 (GAC > AAC, Asp > Asn) and 545 (GAG > AAG, Glu > Lys; GAG > GCG, Glu > Ala). Two of the three *PIK3CA* mutated samples were HPV negative. No mutation in *PIK3CA* gene was detected in DNA samples extracted from CIN3 samples.

## Discussion

Persistent infection with high risk HPVs, the increased expression of E6 and E7 oncoproteins and HPV integration into human DNA, along with chromosomal aberrations and gene alteration at the integration sites, are considered significant features of progression from pre-invasive to invasive cervical cancer [5,35,36]. Few studies, however, have systematically analyzed genetic alterations in HPV-related genital cancers and their significance for cancer staging and as predictive biomarkers.

In this study we observed that 20% of cervical neoplasia cases were mutated in *TP53* gene. The prevalence was highest in adenocarcinoma (36%), followed by squamous cell cervical cancer (16%) and CIN3 (13%). Although the small number of cases analyzed in this study precludes definitive conclusions regarding the absolute difference in mutation rates among the different histological types of cervical cancer groups, the results are in agreement with data obtained from COSMIC databes [10]. However, Ojesina et al. reported similar mutation frequencies (5%) of *TP53* gene and different mutation profiles of other genes in adenocarcinoma and squamous cell carcinoma. Specifically, in squamous cell carcinomas mutations included recurrent E322K substitutions in the MAPK1 gene (8%), inactivating mutations in the HLA-B gene (9%), and mutations in EP300 (16%), FBXW7 (15%), NFE2L2 (4%), *TP53* (5%) and ERBB2 (6%). In adenocarcinoma variations included somatic mutations in ELF3 (13%) and CBFB (8%) genes [18]. On the other hand, the identification of KRAS mutations in 17% of adenocarcinoma and in none of squamous carcinoma samples strongly suggests that they have distinct molecular profiles [28].

The pattern of *TP53* mutations was similar in cervical adenocarcinoma and squamous cell carcinoma and showed an excess of C:G to T:A transitions. This type of mutations, which are the most common in all human cancers, may originate from spontaneous nucleotide changes deriving from deamination of methyl-cytosine [4]. However, it is unclear whether their relative high frequency in cervical cancer may be related to a specific carcinogen or to the excess of oxygen and nitrogen radicals produced by oxidant-generating enzymes during chronic inflammation [37-41]. We found an excess of *TP53* mutations in the mucinous endocervical histotype (54%), however no other study to date addressed the issue of gene mutation distribution across the different subtypes of adenocarcinoma such as endocervical, endometrioid, intestinal, and mixed adenocarcinoma.

Very few studies have analyzed the possible association between mutations in *TP53* gene and prognosis or treatment outcome in adenocarcinoma and squamous cell carcinoma of the cervix. One study has explored whether p53 status, HPV, and LOH on chromosome 3p21.3, 6p21.2, 17p13.1 (on which *TP53* gene is located), and 18q21.2 are associated with prognosis and treatment outcome in 60 patients with squamous cell carcinoma and 5 with adenocarcinoma of the cervix after radiotherapy. Statistical significant differences were observed between tumor size and clinical stage (P = 0.0006), p53 status (P = 0.045), and LOH on 17p13.1 (P = 0.02) [42]. No significant differences in the overall survival and disease-free survival between patients with wild-type p53 and those with mutant p53 were observed in their study. Although, the statistical analysis was hampered by the small number of cases with mutant *TP53* (10.8% of carcinomas).

We analyzed exon 9 of *PIK3CA* gene, encoding for the highly conserved helical domain of the p110alpha catalytic subunit of PI3K, and identified mutations in 11% of adenocarcinoma and 5% of squamous cell cervical cancer (5%) and no mutations in the pre-invasive lesions. These results are in agreement with *PIK3CA* mutation frequencies reported in COSMIC (Catalogue of Somatic Mutations in Cancer) database (11% and 14% in adenocarcinoma and squamous cell carcinoma, respectively) and with those recently obtained by whole exome sequencing analysis of cervical cancer genomes (*PIK3CA* mutation frequencies of 12.5% and 12.6% in adenocarcinoma and squamous cell carcinoma, respectively) [18,43]. Moreover, Cui et al. [44] analyzing exon 1, 9 and 20 of *PIK3CA* gene in cervical carcinomas and CIN3 lesions identified somatic mutations in 8.15% of cervical carcinomas but no mutations in CIN3 cases suggesting that genetic alterations of this proto-oncogene are late events during carcinogenesis. Much higher frequencies of *PIK3CA* mutations, however, were found both in adenocarcinoma (37.5%) and in cervical squamous carcinoma (25%) by mass-spectrometry based analyses [28,45], suggesting either that this technique is much more sensitive than Sanger sequencing or that cancer cases from different populations have different rates of *PIK3CA* mutations.

Interestingly in our study *PIK3CA* gene showed homozygous nucleotide substitutions in all mutated adenocarcinoma suggesting that major genetic alterations affected the locus 3q26.3, where the *PIK3CA* gene is located. Indeed, a recent meta-analysis showed that the most frequent chromosomal aberrations in cervical carcinoma (SCC) occurred as gains (0.55, 95% CI 0.43–0.70) or losses at 3p (0.36, 95% CI 0.27–0.48), specifically at the 3q25-3q29 loci [46]. Genomic gains of locus 3q26 have been associated with the progression from high-grade cervical disease to cancer being detected at lower rate in earlier stages of cervical carcinogenesis and shown to increase with the severity of cervical lesions [47-51]. No study has yet investigated the correlation between the mutation frequency of *PIK3CA* gene and 3q chromosomal aberration frequency.

Mutated *PIK3CA* gene is becoming a promising target for newly discovered anticancer drugs. Janku et al. reported that among patients affected by advanced breast, cervical, endometrial, and ovarian cancers, those with *PIK3CA* mutations treated with PI3K/AKT/mTOR inhibitors demonstrated a higher response rate than patients without mutations [30]. In particular among the 23 *PIK3CA*-mutant patients with breast and gynecologic cancers who experienced treatment failure with standard therapies, was observed a response rate of 30%, which is significantly favorable compared to a 10% response rate in cancer patients with wild type *PIK3CA* treated on the same protocols (P = 0.04) [30]. Moreover, McIntyre et al. reported an association between tumoral *PIK3CA* mutational status and overall survival in patients with cervical cancer treated with radical chemo-radiotherapy. In particular the *PIK3CA* mutation status was strongly associated with overall survival in FIGO stage IB/II patients, unadjusted hazard ratio 6.0 (95% CI 2.1–17.5), p = 0.0002, but not stage III/IVA patients, unadjusted hazard ratio 1.0 (95% CI 0.32–3.1), p = 0.98 [26]. Further prospective clinical trials evaluating patients with *PIK3CA* positive tumors are required to evaluate response to targeted agents such as PI3K inhibitors.

The main limitations of our study include the small sample size and the retrospective design that do not allow the appropriate evaluation of the clinical significance of *TP*53 and *PIK3CA* mutants in terms of overall survival, metastasis-free survival and outcome to therapies. Prospective longitudinal studies are needed to define if the knowledge of *TP53* and/or *PIK3CA* status could provide relevant information for the management of individual patients with different histotypes of adenocarcinoma and squamous cell carcinoma of the cervix.

## Conclusions

In conclusion, our results show distinct mutation profiles in *TP53* and *PIK3CA* genes in cervical adenocarcinoma, squamous cell carcinoma and CIN3. Knowledge of genetic alterations in different cervical cancer histotypes, in addition to currently used viral testing, may provide a basis for future research directions in early diagnostics and personalization of therapy.
